# The Time-Course Changes in Knee Flexion Range of Motion, Muscle Strength, and Rate of Force Development After Static Stretching

**DOI:** 10.3389/fphys.2022.917661

**Published:** 2022-06-02

**Authors:** Masatoshi Nakamura, Yusuke Suzuki, Riku Yoshida, Kazuki Kasahara, Yuta Murakami, Tetsuya Hirono, Satoru Nishishita, Kosuke Takeuchi, Andreas Konrad

**Affiliations:** ^1^ Faculty of Rehabilitation Sciences, Nishi Kyushu University, Kanzaki, Japan; ^2^ Institute for Human Movement and Medical Sciences, Niigata University of Health and Welfare, Niigata, Japan; ^3^ S/PARK Business Planning Group, MIRAI Technology Institute, R&D Integrated Operation Department, Shiseido Co, Ltd., Global Innovation Center, Kanagawa, Japan; ^4^ School of Health and Sport Sciences, Chukyo University, Toyota, Japan; ^5^ Research Fellow of the Japan Society for the Promotion of Science, Tokyo, Japan; ^6^ Institute of Rehabilitation Science, Tokuyukai Medical Corporation, Osaka, Japan; ^7^ Kansai Rehabilitation Hospital, Tokuyukai Medical Corporation, Osaka, Japan; ^8^ Department of Physical Therapy, Faculty of Rehabilitation, Kobe International University, Hyogo, Japan; ^9^ Institute of Human Movement Science, Sport and Health, Graz University, Graz, Austria

**Keywords:** flexibility, knee extensor, maximal voluntary isometric contraction, explosive muscle strength, prolonged effect

## Abstract

Previous studies have shown that longer-duration static stretching (SS) interventions can cause a decrease in muscle strength, especially explosive muscle strength. Furthermore, force steadiness is an important aspect of muscle force control, which should also be considered. However, the time course of the changes in these variables after an SS intervention remains unclear. Nevertheless, this information is essential for athletes and coaches to establish optimal warm-up routines. The aim of this study was to investigate the time course of changes in knee flexion range of motion (ROM), maximal voluntary isometric contraction (MVIC), rate of force development (RFD), and force steadiness (at 5 and 20% of MVIC) after three 60-s SS interventions. Study participants were sedentary healthy adult volunteers (*n* = 20) who performed three 60-s SS interventions of the knee extensors, where these variables were measured before and after SS intervention at three different periods, i.e., immediately after, 10 min, and 20 min the SS intervention (crossover design). The results showed an increase in ROM at all time points (d = 0.86–1.01). MVIC was decreased immediately after the SS intervention (d = −0.30), but MVIC showed a recovery trend for both 10 min (d = −0.17) and 20 min (d = −0.20) after the SS intervention. However, there were significant impairments in RFD at 100 m (*p* = 0.014, F = 6.37, η_p_^2^ = 0.101) and 200 m (*p* < 0.01, F = 28.0, η_p_^2^ = 0.33) up to 20 min after the SS intervention. Similarly, there were significant impairments in force steadiness of 5% (*p* < 0.01, F = 16.2, η_p_^2^ = 0.221) and 20% MVIC (*p* < 0.01, F = 16.0, η_p_^2^ = 0.219) at 20 min after the SS intervention. Therefore, it is concluded that three 60-s SS interventions could increase knee flexion ROM but impair explosive muscle strength and muscle control function until 20 min after the SS intervention.

## Introduction

In sports settings, many coaches and athletes advocate static stretching (SS) during a warm-up routine (Takeuchi et al., 2019). Indeed, many previous studies have shown that a single SS intervention can induce acute increases in range of motion (ROM) (Konrad et al., 2017; Nakamura et al., 2019; Sato et al., 2020). On the other hand, SS interventions of more than 45–60 s are also well known to cause a decrease in muscle strength and explosive performance, which is called “stretch-induced force deficit” (Behm and Chaouachi, 2011; Simic et al., 2013; Behm et al., 2016; Behm et al., 2021).

Although there have been many studies on the acute effects of SS interventions, few studies have investigated the time-course changes of an SS intervention. The time course of changes in stretch-induced force deficit is essential knowledge for athletes and coaches to understand the timing of incorporating an SS intervention during a warm-up routine. However, some previous studies have investigated the time course of changes in stretch-induced force deficit. For example, Mizuno et al. (2014) reported a decrease in maximal voluntary isometric contraction (MVIC) recovered within 10 min after a five 60-s SS intervention of the plantar flexors. Similarly, Ryan et al. (2008b) showed that the plantar flexors’ four 30-s, eight 30-s, and sixteen 30-s SS interventions decreased MVIC significantly, but this was recovered within 10 min. However, Konrad et al. (2019) found a significant decrease in MVIC at 0, 5, and 10 min after an SS intervention of the plantar flexors. However, Budini et al. (2020) showed that MVICs could promote the recovery of muscle spindle sensitivity. Thus, the repeated MVIC measurements in previous studies (Ryan et al., 2008b; Mizuno et al., 2014) could have influenced the muscle strength recovery after the SS interventions. Therefore, it is necessary to investigate the time course of changes after an SS intervention in detail, without including repetitive MVIC measurements, by, for example, measuring every time point in a crossover design.

In addition to MVIC torque, rate of force development (RFD) can be an essential index for explosive muscle strength. Previous studies have shown that an SS intervention can decrease RFD, especially in the late phase of RFD (more than 100 m) (Morais de Oliveira et al., 2012; Trajano et al., 2019; Nakamura et al., 2021b). Andersen and Aagaard. (2006) pointed out that RFD is influenced by different factors in the early (less than 100 m) and late phases (more than 100 m) of isometric contraction (Andersen and Aagaard, 2006). The early phase RFD is mainly influenced by neural drive (Aagaard et al., 2002) and intrinsic muscle contractile properties (Andersen and Aagaard, 2006). During the late phase, MVIC (Andersen and Aagaard, 2006), neural drive (Häkkinen et al., 1985), and the stiffness of the tendinous structures (Bojsen-Møller et al., 2005) are related to late-phase RFD. However, to the best of our knowledge, the time course of changes in RFD after an SS intervention remains unclear. In addition to MVIC and RFD, force steadiness is another important aspect of muscle force control. The force fluctuations during submaximal muscle contractions at a target value torque can be quantified as force steadiness (Tracy, 2007; Hirono et al., 2020; Hirono et al., 2021). A review by Oomen and Van Dieen. (2017) suggested that synchronization is caused by the muscle spindle feedback associated with the small fluctuations in muscle fascicle and spindle length that accompany force variations. Previous studies have reported significant correlations between the force steadiness at 5 and 20% MVIC in healthy young adults (Hirono et al., 2020) and 20% MVIC in older adults (Hirono et al., 2021) and postural control tasks. A few previous studies investigated the effect of SS on force steadiness, and Kato et al. (2011) showed that five 60-s SS interventions could impair force steadiness at 20% MVIC. However, Capobianco et al. (2018) showed no significant change in force steadiness at 20% MVIC 15 min after three 30-s SS interventions. Therefore, it is unclear whether an SS intervention can impair force steadiness. Also, the time course of the changes in force steadiness and the acute effect after an SS intervention have been unclear.

Therefore, this study aimed to investigate the time course of changes in ROM, MVIC, RFD, and force steadiness after three 60-s SS interventions of the knee extensors. Based on the previous studies (Ryan et al., 2008b; Mizuno et al., 2014), we hypothesized that three 60-s SS interventions would impair MVIC, RFD, and force steadiness, but these impairments would be recovered within 20 min.

## Methods

### Experimental Design

**FIGURE 1:**
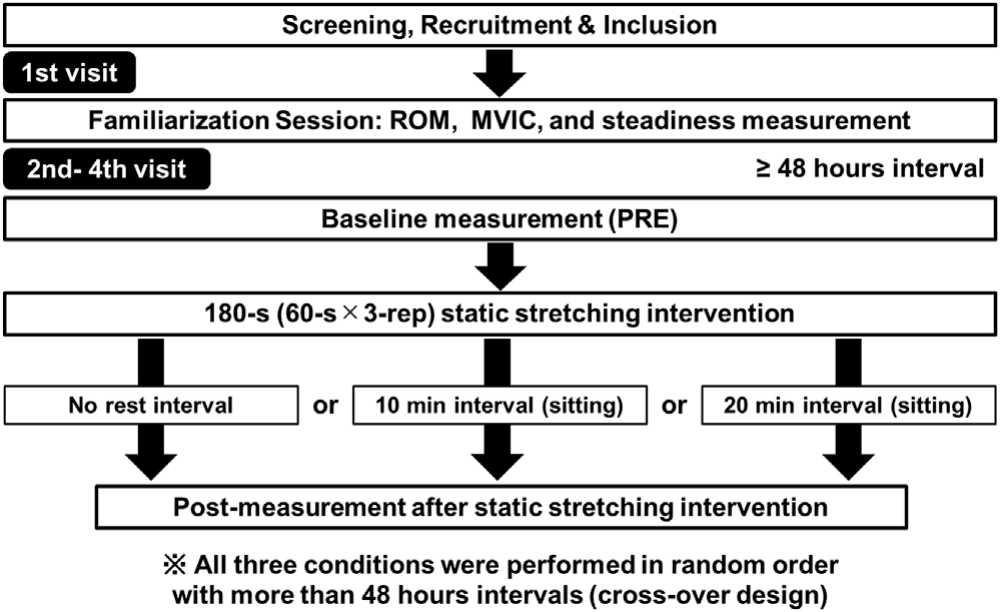
| Experimental protocol. The participants visited the laboratory on four occasions (familiarization session and three experimental sessions, i.e., immediately after (no rest interval), 10-min interval, and 20-min interval after the static stretching intervention). The three experimental sessions were performed in random order with more than 48-h intervals (crossover design).

A randomized repeated-measures experimental design was used to investigate the time course of changes after an SS intervention in knee flexion ROM, MVIC, RFD, and force steadiness of the dominant knee extensors. The dominant leg was defined as the preferred leg for kicking a ball. Participants visited the laboratory on four occasions at intervals of ≥48 h. The first visit was a familiarization trial for the knee flexion ROM, MVIC torque, and force steadiness measurements. On the three subsequent visits, participants were subjected to the following experimental conditions in random order (immediately after, 10 min after, and 20 min after the SS intervention, Figure 1). In the familiarization trials, all participants practiced the knee flexion ROM, MVIC, RFD, and steadiness movements, to ensure comfort and familiarity with the procedures and minimize any potential learning effects. Following previous studies (Hatano et al., 2019; Sato et al., 2020), all outcome variables were measured before (pre), immediately after, 10 min after, and 20 min after the SS intervention. In the 10-min and 20-min conditions, participants remained seated on a chair for the 10 or 20 min after the SS intervention.

### Participants

The sample size required for two-way repeated-measures analysis of variance (ANOVA) [effect size = 0.25 (medium), *α* error = 0.05, and power = 0.95] was calculated using G* power 3.1 software (Heinrich Heine University, Düsseldorf, Germany). The required number of participants was established to be more than 15 participants.

The participants enrolled in this study were 20 sedentary healthy young male volunteers (age 20.5 ± 0.9 years; height 173.2 ± 5.7 cm; body mass 64.1 ± 5.7 kg) who had not performed habitual exercise activities for at least the past 6 months before the assessment. Participants who had a history of neuromuscular disease or musculoskeletal injury in the lower extremity were excluded. All subjects were fully informed of the study procedures and purpose and obtained written informed consent. In this study, we included only male participants to avoid consideration of the potential influence of menstrual cycle variation in women. The Niigata University of Health and Welfare ethics committee approved the study (#18561).

### Knee Flexion Range of Motion

**FIGURE 2:**
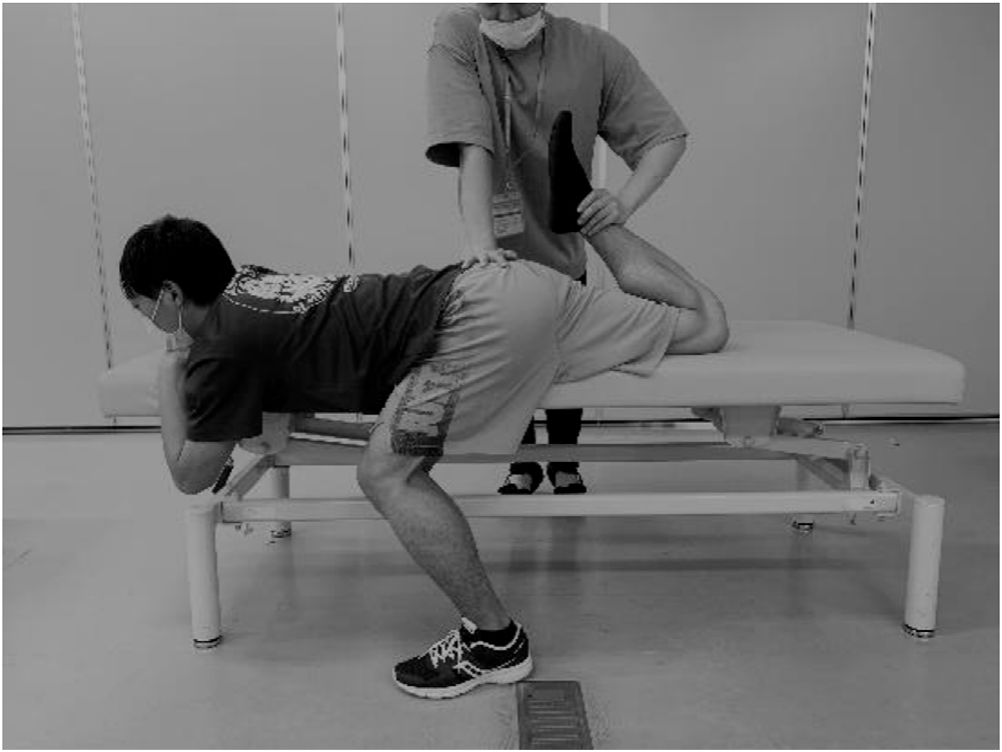
| Knee flexion range of motion (ROM) measurement.

Each participant was placed in a prone position on a massage bed, and the hip flexion of the non-dominant leg was fixed at 120° to prevent movement of the pelvis during the ROM measurements (Figure 2). The investigator brought the dominant leg to full knee flexion, with the hip joint in a neutral position just before the subjects started to feel discomfort or pain (Nakamura et al., 2021a; Takeuchi et al., 2021a). A goniometer was used to measure the knee flexion ROM three times, and the average value was used for further analysis.

### Maximal Voluntary Isometric Contraction Torque and Rate of Force Development Measurement

MVIC torque was measured at a 90° knee angle using the Biodex System 3.0 (Biodex Medical Systems, Shirley, NY, United States). Each participant was seated in the dynamometer chair at an 80° hip flexion angle, with adjustable Velcro straps fixed over the measured limb’s trunk, pelvis, and thigh. After several warm-up submaximal knee extension contractions, the participant was instructed to perform a knee extension as fast and hard as possible and to maintain this knee extension for about 3 s (Ema et al., 2016). The trials were conducted two times, with 60 s of rest between each trial, and the average value of the two MVIC torque measurements was adopted for further analysis. During all the tests, the investigator provided constant verbal encouragement. The intraclass correlation coefficient (ICC) and 95% confidence intervals (CI) were calculated using the data before SS intervention, and the ICC of MVIC was 0.982 (*p* < 0.01, 95% CI = 0.967–0.99).

The torque signals were recorded on a personal computer through an A/D converter operating at 1 kHz (PowerLab16/35, AD Instruments, Australia). The torque signals were also low-pass filtered at 15 Hz using a fourth-order zero-phase lag Butterworth filter (Aagaard et al., 2002; Ema et al., 2016; Nakamura et al., 2021b). After this, the onset of the knee extension was defined as when the torque increased by two standard deviations (SD) above baseline and did not fall below baseline throughout the contraction. The RFD was defined as the slope of the filtered time-torque curve over time intervals of 0–50, 0–100, and 0–200 m from the onset of the knee extension (Aagaard et al., 2002; Ema et al., 2016; Nakamura et al., 2021b). The ICC of RFD at 50, 100, and 200 m were 0.755 (*p* < 0.01, 95% CI = 0.557–0.865), 0.784 (*p* < 0.01, 95% CI = 0.609–0.881), and 0.889 (*p* < 0.01, 95% CI = 0.800–0.939), respectively (*p* < 0.01).

### Force Steadiness Measurements

The force steadiness measurements were performed in a similar way to the MVIC measurements. The torque signals obtained from the dynamometer were sent to the personal computer using the A/D converter with a sampling rate of 1 kHz. The exerted torque was processed with a moving root mean square (RMS) 50-ms time window in real-time, based on previous studies (Hirono et al., 2020; Hirono et al., 2021; Hirono et al., 2022). Based on the MVIC torque of the knee extensors, the target torque values for the force steadiness tasks were set to 5 and 20% of the MVIC for individual participants. In addition, to account for the stretch-induced force deficit after the SS intervention measurements, we recalculated the 5 and 20% target torque based on the MVIC torque in the post-SS intervention. Each force steadiness task was performed twice for the pre-measurements and one time after the SS intervention periods. Each force steadiness task was performed, in random order, with a 60-s rest interval. The target and exerted torques were shown on the personal computer’s monitor for visual feedback during the force steadiness tasks. The participant was instructed to exert torque for 25 s, which included a duration of 10-s, where the torque was gradually increased from the baseline torque to the target torque and then stabilized at this target value (Hirono et al., 2020; Hirono et al., 2021; Hirono et al., 2022). Therefore, the first 10 s of torque data were omitted to ensure steady readings. The force steadiness was identified as the coefficient of variation (CV) of the knee extension torque, i.e., 100*SD/mean (%) using the last 15 s of exerted torque data. The average CV of the two trials for each force steadiness task in the pre-measurement was used for the analysis. A high CV for the force steadiness value indicated more force oscillation (i.e., the ability to control force exertion is lower). The ICC of CV values at 5 and 20% were 0.646 (*p* < 0.01, 95% CI = 0.358–0.805), and 0.727 (*p* < 0.01, 95% CI = 0.686–0.904), respectively (*p* < 0.01).

### Static Stretching Intervention

The SS intervention was performed in a similar way to the knee flexion ROM assessment (Figure 2). Three 60-s stretching interventions with 30-s intervals were performed (Nakamura et al., 2020; Takeuchi et al., 2021a). The stretching intensity (angle) was defined as just before the subjects started to feel discomfort or pain (Takeuchi et al., 2021a; Nakamura et al., 2021b). Participants were instructed to relax their bodies during the stretching intervention.

### Statistical Analysis

SPSS (version 24.0; SPSS Japan Inc. Tokyo, Japan) was used for the statistical analysis. The distribution of the data was assessed using a Shapiro-Wilk test, and it was confirmed that the data followed a normal distribution. For all the variables, a two-way repeated-measures ANOVA using two factors [test time (before vs. after SS) and condition (immediately after SS vs. 10 vs. 20 min)] was used to analyze the interaction and main effect. Classification of effect size (ES) was set where the effect size of split-plot ANOVA (η_p_^2^) was deemed to be small (<0.01); an η_p_^2^ of 0.02–0.1 was deemed to be medium, and an η_p_^2^ greater than 0.1 was deemed to be large (Cohen 1988). Also, we calculated ES (d) as the mean difference between the pre-and post-values divided by the pooled pre- and post-SS intervention SD values. An ES of 0.00–0.19 was considered trivial, 0.20–0.49 was considered small, 0.50–0.79 was considered moderate, and ≥0.80 was considered large (Cohen, 1988). The significance level was set to 5%, and all the results are shown as mean ± SD.

## Results

Table 1 lists the results for the knee flexion ROM, MVIC torque, RFD, and CV values. The two-way repeated-measures ANOVA indicated no significant interactions for all the variables. However, there were main effects for test time for the knee flexion ROM (F = 65.3, *p* < 0.01, η_p_^2^ = 0.534) and MVIC (F = 16.1, *p* < 0.01, η_p_^2^ = 0.22). The main effect results showed that three 60-s SS interventions could increase knee flexion ROM and decrease MVIC up to 20 min after SS intervention. MVIC was decreased immediately after the SS intervention (d = −0.30), but MVIC showed a recovery trend for both 10 min (d = −0.17) and 20 min (d = −0.20) after the SS intervention.

**TABLE 1:** Changes (mean ± SD) in knee flexion range of motion (ROM), maximal voluntary isometric contraction (MVIC) torque of the knee extensors, rate of force development (RFD) at 50, 100, and 200 m, and coefficient of variation (CV) values at 5 and 20% MVIC before (pre) and immediately after, 10 min after, and 20 min after the static stretching (SS) intervention. The two-way ANOVA results (T: test time effect, T × C: test time × conditions interaction effect; F-value) and partial η^2^ (η_p_^2^) are shown in the right column.

	Immediately after SS		10-min after SS		20-min after SS		ANOVA results
	Pre	After SS	Pre	10-min	Pre	20-min	*p* value, F value, η_p_^2^
Knee flexion ROM (°)	139.5 ± 11.7	149.0 ± 7.2	143.5 ± 10.4	150.3 ± 5.4	142.3 ± 9.9	149.8 ± 5.1	**T**: *p* < 0.01, F = 65.3, η_p_^2^ = 0.534
Effect size	Δ = 7.2 ± 5.8%	d = 1.01	Δ = 5.1 ± 6.3%	d = 0.86	Δ = 5.6 ± 5.6%	d = 1.00	**T x C**: *p* = 0.500, F = 0.70, η_p_^2^ = 0.024
MVIC (Nm)	169.7 ± 31.2	160.6 ± 29.5	167.0 ± 28.9	162.1 ± 29.1	167.1 ± 30.0	161.2 ± 29.4	**T**: *p* < 0.01, F = 16.1, η_p_^2^ = 0.22
Effect size	Δ = −5.2 ± 5.8%	d = −0.30	Δ = −2.7 ± 8.0%	d = -0.17	Δ = −3.2 ± 9.5%	d = −0.20	**T x C**: *p* = 0.556, F = 0.60, η_p_^2^ = 0.02
RFD at 50 m (Nm/ms)	0.48 ± 0.20	0.42 ± 0.20	0.55 ± 0.22	0.48 ± 0.18	0.49 ± 0.28	0.48 ± 0.23	**T**: *p* = 0.055, F = 3.85, η_p_^2^ = 0.063
Effect size	Δ = −4.5 ± 38.1%	d = −0.29	Δ = −1.9 ± 56.7%	d = −0.38	Δ = 16.3 ± 72.2%	d = −0.06	**T x C**: *p* = 0.903, F = 0.51, η_p_^2^ = 0.018
RFD at 100 m (Nm/ms)	0.56 ± 0.19	0.49 ± 0.17	0.58 ± 0.20	0.51 ± 0.17	0.56 ± 0.24	0.52 ± 0.19	**T**: *p* = 0.014, F = 6.37, η_p_^2^ = 0.101
Effect size	Δ = −6.1 ± 35.8%	d = −0.39	Δ = −4.2 ± 42.5%	d = −0.36	Δ = −0.5 ± 35.2%	d = −0.19	**T x C**: *p* = 0.852, F = 0.16, η_p_^2^ = 0.006
RFD at 200 m (Nm/ms)	0.50 ± 0.12	0.43 ± 0.13	0.52 ± 0.12	0.45 ± 0.10	0.51 ± 0.15	0.46 ± 0.13	**T**: *p* < 0.01, F = 28.0, η_p_^2^ = 0.33
Effect size	Δ = −13.2 ± 16.1%	d = −0.57	Δ = −10.8 ± 22.4%	d = −0.63	Δ = −8.4 ± 14.8%	d = −0.36	**T x C**: *p* = 0.732, F = 0.314, η_p_^2^ = 0.011
CV values at 5% MVIC (%)	1.28 ± 0.39	1.67 ± 0.87	1.31 ± 0.42	1.46 ± 0.46	1.23 ± 0.37	1.55 ± 0.53	**T**: *p* < 0.01, F = 16.2, η_p_^2^ = 0.221
Effect size	Δ = 34.9 ± 54.6%	d = 0.62	Δ = 14.1 ± 27.3%	d = 0.33	Δ = 25.4 ± 25.8%	d = 0.70	**T x C**: *p* = 0.375, F = 0.999, η_p_^2^ = 0.034
CV values at 20% MVIC (%)	1.87 ± 0.54	2.22 ± 0.96	1.89 ± 0.63	2.12 ± 0.87	1.85 ± 0.67	2.15 ± 0.76	**T**: *p* < 0.01, F = 16.0, η_p_^2^ = 0.219
Effect size	Δ = 19.3 ± 35.9%	d = 0.47	Δ = 12.2 ± 20.1%	d = 0.31	Δ = 19.1 ± 25.0%	d = 0.42	**T x C**: *p* = 0.803, F = 0.22, η_p_^2^ = 0.008

In addition, there was no main effect for RFD at 50 m, but significant main effects for RFD at 100 and 200 m, and CV values at 5 and 20% MVIC. The main effect results showed significant impairments in RFD at 100 and 200 m up to 20 min after the SS intervention. Similarly, there were significant impairments in CV values at 5 and 20% MVIC at 20 min after the SS intervention.

## Discussion

This study investigated the time-course changes after three 60-s SS interventions in knee flexion ROM, MVIC, RFD, and force steadiness. We conducted the measurements on different days and not on the same day to exclude the effect of the measurement itself on outcome variables. To the best of our knowledge, this is the first study to have investigated the time course effect of SS intervention on knee flexion ROM and muscle strength, including explosive muscle strength (i.e., RFD) and muscle force control function (i.e., force steadiness) of the knee extensors. The results showed that three 60-s SS interventions could decrease MVIC, but MVIC recovered after 10 min. However, the RFD at 200 m remained significantly decreased until 20 min after the SS intervention. In addition, force steadiness showed a decreasing trend, with a significant decrease seen 20 min after the SS intervention. These results indicate that the stretch-induced force deficit caused by the three 60-s SS interventions recovers after 10 min in the knee extensors. On the other hand, RFD decreased, and force steadiness increased 20 min after the SS intervention, suggesting that an SS intervention alone should be avoided before events that require explosive strength and muscle force control function.

The results showed that three 60-s SS interventions could increase knee flexion ROM significantly, and the increase in knee flexion ROM was sustained 20 min after the SS intervention. These results were consistent with the previous studies (Mizuno et al., 2013b; Hatano et al., 2019). Previous studies have reported that a 3-min or 5-min SS intervention can increase dorsiflexion ROM, but a decrease in muscle stiffness returns to baseline in the first few minutes (Konrad et al., 2019; 2020). Previous studies have also shown that three 60-s SS interventions can decrease the stiffness of the knee extensors (Nakamura et al., 2020; Takeuchi et al., 2021a). However, other studies have shown that a change in viscoelastic property is recovered within 10–15 min (Ryan et al., 2008a; Mizuno et al., 2013a; Mizuno et al., 2013b). Thus, the detailed mechanism for the increase of knee flexion ROM remains unclear, but the change in muscle stiffness can be attributed to the increase in knee flexion ROM. The other mechanism in the increase of knee flexion ROM can be attributed to changes in stretch sensation (Mizuno et al., 2013b; Nakamura et al., 2021a). Thus, both changes in muscle stiffness and/or stretch tolerance can result in an increase in knee flexion ROM 20 min after an SS intervention.

Our results showed that the three 60-s SS interventions decreased the MVIC of the knee extensors (d = −0.30), but the decrease in MVIC could show a recovering trend within 10 min after the SS intervention (d = −0.17). These results support the findings of previous studies, which showed that stretch-induced force deficit could recover within 10 min (Ryan et al., 2008b; Mizuno et al., 2014). However, one big issue is that the MVIC measurements were repeated in the previous studies (Ryan et al., 2008b; Mizuno et al., 2014). SS can induce modifications in the persistent inward currents (PICs) (Behm et al., 2021) and alterations of muscle spindle sensitivity (Budini et al., 2020), which both adversely affect muscle activation. As described above, Budini et al. (2020) showed that MVIC can promote the recovery of muscle spindle sensitivity. Thus, in the previous studies (Ryan et al., 2008b; Mizuno et al., 2014), repetitive MVIC measurements might have promoted the recovery from an MVIC decrease after SS intervention, resulting in recovery within 10 min. Therefore, in this study, we investigated the time-course changes in MVIC on a different day to minimize the effect of repetitive MVIC measurements. However, this study revealed that MVIC could show a recovering trend within 10 min after the three 60-s SS interventions, consistent with these previous studies (Ryan et al., 2008b; Mizuno et al., 2014). PICs are depolarizing currents generated by voltage-sensitive sodium and calcium channels predominantly located on the motoneurons’ dendrites (Heckman et al., 2005). Also, PICs are a fundamental component of standard motor output observed in humans, and reductions in PIC amplitude can significantly affect the ability to exert muscle strength (Trajano et al., 2020). Trajano et al. (2014) showed that PICs partially recover at 5 min after SS and fully recover at 10 min after SS (Trajano et al., 2014). Thus, taken together with the previous studies and the results of this study, MVIC might recover within 10 min after the three 60-s SS interventions due to the modifications in the PICs.

The results obtained in this study showed no significant changes in early phase RFD, i.e., RFD at 50 m, but a large decrease in RFD at 200 m immediately after the SS intervention, which is consistent with previous studies (Morais de Oliveira et al., 2012; Trajano et al., 2019; Nakamura et al., 2021b). Interestingly, we expanded the knowledge of these previous studies, and our results showed that RFD at 200 m significantly decreased 20 min after the SS intervention. Andersen and Aagaard. (2006) pointed out that RFD is influenced by different factors in the early (less than 100 m) and late phases (more than 100 m) of isometric contraction (Andersen and Aagaard, 2006). During the late phase, MVIC (Andersen and Aagaard, 2006), neural drive (Häkkinen et al., 1985), and the stiffness of the tendinous structures (Bojsen-Møller et al., 2005) are related to RFD. In this study, MVIC recovered within 10 min after the SS intervention, and the changes in neural drive and/or stiffness of the tendon-aponeurosis complex could affect the RFD at 200 m, 20 min after the SS intervention. In a range of sports involving explosive movements (e.g., sprinting, jumping), the time allowed to exert force is typically very limited (from 50 to 250 m). Therefore, the results of this study showed that MVIC recovered, but RFD at 200 m continued to decrease up to 20 min after the SS intervention, which should be noted for events that require explosive muscle strength.

The results of this study showed that CV values at 5 and 20% MVIC were increased, i.e., force steadiness was impaired up to 20 min after the SS intervention, which is consistent with the previous study by Kato et al. (2011), and expanded the results. Specifically, force production is regulated by two main parameters: the recruitment of motor units and the motor unit firing behavior (motor unit firing rate, firing rate variability, motor unit firing synchronization, and common drive to motor units) (Hu et al., 2013; Hu et al., 2014). De Luca et al. (2009) suggest that the ongoing activity of muscle spindles likely influences the degree of the common drive during a contraction. In addition, an SS intervention can induce alterations in muscle spindle sensitivity (Budini et al., 2020). The detailed mechanism is unclear, but the alterations in the recruitment of motor units and the motor unit firing behavior via changes in muscle spindle sensitivity could impair the force control function (i.e., force steadiness). Carville et al. (2007) showed that older adults who have a history of falling have a significantly larger value in force steadiness during knee extension than those who have not experienced falls. The results suggested that older adults who have a history of falling are a lower ability to control force exertion. Furthermore, previous studies reported significant correlations between force steadiness at 5 and 20% MVIC in healthy young adults (Hirono et al., 2020) and 20% MVIC in older adults (Hirono et al., 2021) and postural control tasks. Therefore, an SS intervention could impair the force control function, and the impaired force control function could decrease the balance function 20 min after the SS intervention.

The present study has some limitations. First, the participants were not athletes or older adults but sedentary male volunteers. Second, unlike actual sports situations, we investigated the effects of SS intervention alone. In the future, it is necessary to investigate the effects of SS intervention when it is incorporated into actual warm-up routines for athletes and older adults.

## Conclusion

We investigated the time-course changes after the three 60-s SS interventions in the knee extensors, and our results showed that the increasing knee flexion ROM continued until 20 min after the SS intervention, but the MVIC decrease tended to be restored after 10 min. Therefore, it may be effective to incorporate an SS intervention early on in a warm-up routine if the goal is to increase ROM. On the other hand, RFD at 200 m and force steadiness was impaired up to 20 min after the SS intervention. Therefore, an SS intervention should be avoided in sports requiring explosive muscle strength and balance function. On the other hand, it has been reported that a dynamic warm-up (Samson, et al., 2012; Reid, JC et al., 2018), aerobic exercise (Takeuchi and Nakamura, 2020; Takeuchi et al., 2021b), or foam rolling with and without vibration (Nakamura et al., 2022) after an SS intervention has a potentiation effect for the stretch-induced force deficit. Thus, it will be necessary to establish an optimal warm-up routine by investigating the effects of an SS intervention in actual warm-up routines.

## Data Availability

The raw data supporting the conclusions of this article will be made available by the authors, without undue reservation.

## References

[B1] Aagaard P. Simonsen E. B. Andersen J. L. Magnusson P. Dyhre-Poulsen P. (2002). Increased Rate of Force Development and Neural Drive of Human Skeletal Muscle Following Resistance Training. J. Appl. Physiology 93, 1318–1326. 10.1152/japplphysiol.00283.2002 12235031

[B2] Andersen L. L. Aagaard P. (2006). Influence of Maximal Muscle Strength and Intrinsic Muscle Contractile Properties on Contractile Rate of Force Development. Eur. J. Appl. Physiol. 96, 46–52. 10.1007/s00421-005-0070-z 16249918

[B3] Behm D. G. Blazevich A. J. Kay A. D. McHugh M. (2016). Acute Effects of Muscle Stretching on Physical Performance, Range of Motion, and Injury Incidence in Healthy Active Individuals: a Systematic Review. Appl. Physiol. Nutr. Metab. 41, 1–11. 10.1139/apnm-2015-0235 26642915

[B4] Behm D. G. Chaouachi A. (2011). A Review of the Acute Effects of Static and Dynamic Stretching on Performance. Eur. J. Appl. Physiol. 111, 2633–2651. 10.1007/s00421-011-1879-2 21373870

[B5] Behm D. G. Kay A. D. Trajano G. S. Blazevich A. J. (2021). Mechanisms Underlying Performance Impairments Following Prolonged Static Stretching without a Comprehensive Warm-Up. Eur. J. Appl. Physiol. 121, 67–94. 10.1007/s00421-020-04538-8 33175242

[B6] Bojsen-Møller J. Magnusson S. P. Rasmussen L. R. Kjaer M. Aagaard P. (2005). Muscle Performance during Maximal Isometric and Dynamic Contractions Is Influenced by the Stiffness of the Tendinous Structures. J. Appl. Physiol. (1985) 99, 986–994. 10.1152/japplphysiol.01305.2004 15860680

[B7] Budini F. Rafolt D. Christova M. Gallasch E. Tilp M. (2020). The Recovery of Muscle Spindle Sensitivity Following Stretching Is Promoted by Isometric but Not by Dynamic Muscle Contractions. Front. Physiol. 11, 905. 10.3389/fphys.2020.00905 32848855 PMC7418680

[B8] Capobianco R. A. Almuklass A. M. Enoka R. M. (2018). Manipulation of Sensory Input Can Improve Stretching Outcomes. Eur. J. Sport Sci. 18 (1), 83–91. 10.1080/17461391.2017.1394370 29105593

[B9] Carville S. F. Perry M. C. Rutherford O. M. Smith I. C. H. Newham D. J. (2007). Steadiness of Quadriceps Contractions in Young and Older Adults with and without a History of Falling. Eur. J. Appl. Physiol. 100, 527–533. 10.1007/s00421-006-0245-2 16983499

[B10] Cohen J. (1988). Statistical Power Analysis for the Behavioral Sciences. Hillsdale: Routledge.

[B11] De Luca C. J. Gonzalez-Cueto J. A. Bonato P. Adam A. (2009). Motor Unit Recruitment and Proprioceptive Feedback Decrease the Common Drive. J. Neurophysiology 101, 1620–1628. 10.1152/jn.90245.2008 PMC2666397 18562556

[B12] Ema R. Saito M. Ohki S. Takayama H. Yamada Y. Akagi R. (2016). Association between Rapid Force Production by the Plantar Flexors and Balance Performance in Elderly Men and Women. Age 38, 475–483. 10.1007/s11357-016-9949-3 27581165 PMC5266226

[B13] Häkkinen K. Alén M. Komi P. V. (1985). Changes in Isometric Force- and Relaxation-Time, Electromyographic and Muscle Fibre Characteristics of Human Skeletal Muscle during Strength Training and Detraining. Acta Physiol. Scand. 125, 573–585. 4091001 10.1111/j.1748-1716.1985.tb07760.x

[B14] Hatano G. Suzuki S. Matsuo S. Kataura S. Yokoi K. Fukaya T. (2019). Hamstring Stiffness Returns More Rapidly after Static Stretching Than Range of Motion, Stretch Tolerance, and Isometric Peak Torque. J. Sport Rehabil. 28, 325–331. 10.1123/jsr.2017-0203 29252096

[B15] Heckman C. J. Gorassini M. A. Bennett D. J. (2005). Persistent Inward Currents in Motoneuron Dendrites: Implications for Motor Output. Muscle Nerve 31 (2), 135–156. 10.1002/mus.20261 15736297

[B16] Hirono T. Ikezoe T. Taniguchi M. Yamagata M. Miyakoshi K. Umehara J. (2020). Relationship between Ankle Plantar Flexor Force Steadiness and Postural Stability on Stable and Unstable Platforms. Eur. J. Appl. Physiol. 120, 1075–1082. 10.1007/s00421-020-04346-0 32172293

[B17] Hirono T. Ikezoe T. Taniguchi M. Yamagata M. Umehara J. Ichihashi N. (2022). Acute Effects of Ankle Plantar Flexor Force-Matching Exercises on Postural Strategy during Single Leg Standing in Healthy Adults. Gait Posture 92, 428–434. 10.1016/j.gaitpost.2021.12.021 34979429

[B18] Hirono T. Ikezoe T. Yamagata M. Kato T. Kimura M. Ichihashi N. (2021). Relationship between Postural Sway on an Unstable Platform and Ankle Plantar Flexor Force Steadiness in Community-Dwelling Older Women. Gait Posture 84, 227–231. 10.1016/j.gaitpost.2020.12.023 33383532

[B19] Hu X. Rymer W. Z. Suresh N. L. (2014). Motor Unit Firing Rate Patterns during Voluntary Muscle Force Generation: a Simulation Study. J. Neural Eng. 11, 026015. 10.1088/1741-2560/11/2/026015 24658323

[B20] Hu X. Rymer W. Z. Suresh N. L. (2013). Motor Unit Pool Organization Examined via Spike-Triggered Averaging of the Surface Electromyogram. J. Neurophysiology 110, 1205–1220. 10.1152/jn.00301.2012 PMC4073930 23699053

[B21] Kato E. Vieillevoye S. Balestra C. Guissard N. Duchateau J. (2011). Acute Effect of Muscle Stretching on the Steadiness of Sustained Submaximal Contractions of the Plantar Flexor Muscles. J. Appl. Physiology 110 (2), 407–415. 10.1152/japplphysiol.01087.2010 21127213

[B22] Konrad A. Reiner M. M. Thaller S. Tilp M. (2019). The Time Course of Muscle-Tendon Properties and Function Responses of a Five-Minute Static Stretching Exercise. Eur. J. Sport Sci. 19, 1195–1203. 10.1080/17461391.2019.1580319 30821657 PMC6816483

[B23] Konrad A. Stafilidis S. Tilp M. (2017). Effects of Acute Static, Ballistic, and PNF Stretching Exercise on the Muscle and Tendon Tissue Properties. Scand. J. Med. Sci. Sports 27, 1070–1080. 10.1111/sms.12725 27367916 PMC5479471

[B24] Konrad A. Tilp M. (2020). The Time Course of Muscle-Tendon Unit Function and Structure Following Three Minutes of Static Stretching. J. Sports Sci. Med. 19 (1), 52–28. 32132827 PMC7039016

[B25] Mizuno T. Matsumoto M. Umemura Y. (2013a). Decrements in Stiffness Are Restored within 10 Min. Int. J. Sports Med. 34, 484–490. 10.1055/s-0032-1327655 23143704

[B26] Mizuno T. Matsumoto M. Umemura Y. (2014). Stretching-induced Deficit of Maximal Isometric Torque Is Restored within 10 minutes. J. Strength Cond. Res. 28, 147–153. 10.1519/jsc.0b013e3182964220 23615480

[B27] Mizuno T. Matsumoto M. Umemura Y. (2013b). Viscoelasticity of the Muscle-Tendon Unit Is Returned More Rapidly Than Range of Motion after Stretching. Scand. J. Med. Sci. Sports 23, 23–30. 10.1111/j.1600-0838.2011.01329.x 21564309

[B28] Morais de Oliveira A. L. Greco C. C. Molina R. Denadai B. S. (2012). The Rate of Force Development Obtained at Early Contraction Phase Is Not Influenced by Active Static Stretching. J. Strength Cond. Res. 26, 2174–2179. 10.1519/jsc.0b013e31823b0546 21997454

[B29] Nakamura M. Sato S. Kiyono R. Takahashi N. Yoshida T. (2019). Effect of Rest Duration between Static Stretching on Passive Stiffness of Medial Gastrocnemius Muscle *In Vivo* . J. Sport Rehabil. 1-16, 1–16. 10.1123/jsr.2018-0376 31094610

[B30] Nakamura M. Sato S. Kiyono R. Yahata K. Yoshida R. Fukaya T. A. (2021a). Comparison of the Acute Effects of Hold-Relax and Static Stretching Among Older Adults. Biol. (Basel) 10, 126. 10.3390/biology10020126 PMC7914644 33562673

[B31] Nakamura M. Sato S. Murakami Y. Kiyono R. Yahata K. Sanuki F. (2020). The Comparison of Different Stretching Intensities on the Range of Motion and Muscle Stiffness of the Quadriceps Muscles. Front. Physiol. 11, 628870. 10.3389/fphys.2020.628870 33519530 PMC7838703

[B32] Nakamura M. Konrad A. Kasahara K. Yoshida R. Murakami Y. Sato S. (2022). The Combined Effect of Static Stretching and Foam Rolling with or without Vibration on the Range of Motion, Muscle Performance, and Tissue Hardness of the Knee Extensor. J. Strength Cond. Res. 10.1519/jsc.0000000000004263 PMC7614110 35544351

[B33] Nakamura M. Sato S. Kiyono R. Yahata K. Yoshida R. Fukaya T. (2021b). Relationship between Changes in Passive Properties and Muscle Strength after Static Stretching. J. Bodyw. Mov. Ther. 28, 535–539. 10.1016/j.jbmt.2021.09.012 34776191

[B34] Oomen N. M. C. W. van Dieën J. H. (2017). Effects of Age on Force Steadiness: A Literature Review and Meta -analysis. Ageing Res. Rev. 35, 312–321. 10.1016/j.arr.2016.11.004 27836706

[B35] Reid J. C. Greene R. Young J. D. Hodgson D. D. Blazevich A. J. Behm D. G. (2018). The Effects of Different Durations of Static Stretching within a Comprehensive Warm-Up on Voluntary and Evoked Contractile Properties. Eur. J. Appl. Physiol. 118 (7), 1427–1445. 10.1007/s00421-018-3874-3 29721606

[B36] Ryan E. D. Beck T. W. Herda T. J. Hull H. R. Hartman M. J. Costa P. B. (2008a). The Time Course of Musculotendinous Stiffness Responses Following Different Durations of Passive Stretching. J. Orthop. Sports Phys. Ther. 38, 632–639. 10.2519/jospt.2008.2843 18827325

[B37] Ryan E. D. Beck T. W. Herda T. J. Hull H. R. Hartman M. J. Stout J. R. (2008b). Do practical Durations of Stretching Alter Muscle Strength? A Dose-Response Study. Med. Sci. Sports Exerc. 40, 1529–1537. 10.1249/mss.0b013e31817242eb 18614936

[B38] Samson M. Button D. C. Chaouachi A. Behm D. G. (2012). Effects of Dynamic and Static Stretching within General and Activity Specific Warm-Up Protocols. J. Sports Sci. Med. 111 (2), 279–285. PMC3737866 24149201

[B39] Sato S. Kiyono R. Takahashi N. Yoshida T. Takeuchi K. Nakamura M. (2020). The Acute and Prolonged Effects of 20-s Static Stretching on Muscle Strength and Shear Elastic Modulus. PLoS One 15, e0228583. 10.1371/journal.pone.0228583 32027694 PMC7004320

[B40] Simic L. Sarabon N. Markovic G. (2013). Does Pre-exercise Static Stretching Inhibit Maximal Muscular Performance? A Meta-Analytical Review. Scand. J. Med. Sci. Sports 23, 131–148. 10.1111/j.1600-0838.2012.01444.x 22316148

[B41] Takeuchi K. Nakamura M. (2020). Influence of Aerobic Exercise after Static Stretching on Flexibility and Strength in Plantar Flexor Muscles. Front. Physiol. 11, 612967. 10.3389/fphys.2020.612967 33424636 PMC7793924

[B42] Takeuchi K. Nakamura M. Kakihana H. Tsukuda F. (2019). A Survey of Static and Dynamic Stretching Protocol. Int. J. Sport Health Sci. 17, 72–79. 10.5432/ijshs.201829

[B43] Takeuchi K. Sato S. Kiyono R. Yahata K. Murakami Y. Sanuki F. (2021a). High-Intensity Static Stretching in Quadriceps Is Affected More by its Intensity Than its Duration. Front. Physiol. 12, 709655. 10.3389/fphys.2021.709655 34290625 PMC8287525

[B44] Takeuchi K. Takemura M. Nakamura M. Tsukuda F. Miyakawa S. (2021b). The Effects of Using a Combination of Static Stretching and Aerobic Exercise on Muscle Tendon Unit Stiffness and Strength in Ankle Plantar-Flexor Muscles. Eur. J. Sport Sci. 1-7, 18. 10.1080/17461391.2020.1866079 33331805

[B45] Tracy B. L. (2007). Force Control Is Impaired in the Ankle Plantarflexors of Elderly Adults. Eur. J. Appl. Physiol. 101, 629–636. 10.1007/s00421-007-0538-0 17701201

[B46] Trajano G. S. Taylor J. L. Orssatto L. B. R. McNulty C. R. Blazevich A. J. (2020). Passive Muscle Stretching Reduces Estimates of Persistent Inward Current Strength in Soleus Motor Units. J. Exp. Biol. 223 (223), jeb229922. 10.1242/jeb.229922 32978317

[B47] Trajano G. S. Seitz L. B. Nosaka K. Blazevich A. J. (2014). Can Passive Stretch Inhibit Motoneuron Facilitation in the Human Plantar Flexors? J. Appl. Physiology 117, 1486–1492. 10.1152/japplphysiol.00809.2014 25342705

[B48] Trajano G. S. Seitz L. B. Nosaka K. Blazevich A. J. (2019). Passive Muscle Stretching Impairs Rapid Force Production and Neuromuscular Function in Human Plantar Flexors. Eur. J. Appl. Physiol. 119, 2673–2684. 10.1007/s00421-019-04244-0 31650306

